# Integration of machine learning and large language models for screening and identifying key risk factors of acute kidney injury after cardiac surgery

**DOI:** 10.3389/fmed.2025.1618222

**Published:** 2025-11-06

**Authors:** Zishan Li, Lei Wang, Xunying Zhang, Aiping Wu, Tao Liu

**Affiliations:** 1 College of Science, North China University of Science and Technology, Tangshan, China; 2 Department of Urology, North China University of Science and Technology Affiliated Hospital, Tangshan, China; 3 College of Automotive Engineering, Hebei College of Science and Technology, Tangshan, China; 4 School of Basic Medical Sciences, North China University of Science and Technology, Tangshan, China

**Keywords:** acute kidney injury (AKI), large language models (LLMs), lasso regression, random forest, MIMIC-IV database

## Abstract

**Objectives:**

This study aimed to identify critical risk factors for acute kidney injury (AKI) following cardiac surgery. By integrating patient data from the MIMIC-IV database with large language models (LLMs) and machine learning algorithms, we ensured the clinical relevance of the selected risk factors, providing robust insights for the early identification and intervention of postoperative AKI.

**Methods:**

Intensive care unit (ICU) data of patients from the MIMIC-IV database undergoing cardiac surgery were analyzed. Lasso regression and random forest algorithms were used to select significant predictive features from high-dimensional data. Model evaluation involved 10-fold cross-validation and metrics including accuracy, sensitivity, specificity, and the area under the curve. To enhance clinical relevance, LLMs-simulated expert judgment in cardiology and nephrology, which was further validated through discussions with clinical experts.

**Results:**

In the cohort consisting of 4,565 patients, a total of 113 important and shared risk factors for AKI were identified, including variables such as anion gap, arterial partial pressure of oxygen (PaO_2_), and fraction of inspired oxygen (FiO_2_). Among these, 18 key variables were identified as postoperative AKI predictors via machine learning and LLMs-simulated expert validation. These included anchor age, Creatinine (serum), BUN (Blood Urea Nitrogen), Potassium (serum), Sodium (serum), Lactic Acid, Troponin-T, Furosemide (Lasix), Vancomycin (Random), Gentamicin (Trough), Albumin 5%, ART BP Mean, Cardiac Output (thermodilution), Brain Natriuretic Peptide (BNP), Absolute Count - Lymphs, Absolute Count - Monos, and Absolute Count - Neuts. The integration of LLMs with machine learning algorithms proved effective in accurately identifying clinically relevant risk factors.

**Conclusion:**

The proposed risk prediction approach for postoperative AKI following cardiac surgery, based on the collaborative analysis of machine learning and large language models (LLMs), effectively identified and validated key clinical risk factors. By simulating expert clinical reasoning, the LLMs significantly enhanced the medical relevance of feature selection and improved the clinical interpretability of the model. This approach provides a solid theoretical and practical foundation for the precise early identification and clinical intervention of postoperative AKI in cardiac surgery patients.

## 1 Introduction

Acute kidney injury (AKI) is a common and serious clinical syndrome, especially among patients in the intensive care unit (ICU) (1). The occurrence of AKI not only significantly increases the patient’s hospitalization time and medical expenses, but is also closely related to high short-term and long-term mortality. According to statistics, the incidence of AKI in ICU patients can be as high as 40%–60%, with about 10%–15% of these patients requiring renal replacement therapy (RRT). Therefore, effectively preventing, diagnosing, and intervening in AKI promptly is of great significance for improving the early identification rate of AKI after cardiac surgery and optimizing clinical intervention (2).

Currently, the diagnosis of AKI mainly relies on the increase in serum creatinine (SCr) levels and the decrease in urine volume (3). However, these traditional indicators have certain limitations. First, the increase in SCr usually lags behind the actual damage to renal function, resulting in the early identification of AKI not being timely enough. Second, urine volume is affected by many factors, such as fluid management and the use of diuretics such as furosemide, and it is challenging to accurately assess urine volume. In addition, traditional AKI risk scoring systems (such as the SOFA score and SAPS score) are based on linear regression model, which are difficult to fully capture complex non-linear relationships and high-dimensional data features (4). In recent years, the application of LLMs (large-scale language model) and machine learning technology in the medical field has gradually increased, especially in disease prediction and diagnosis (5). These technologies can effectively process and analyze structured data, mine complex patterns and potential relationships within the data, and thus provide support for clinical decision-making. Therefore, this study used LASSO and random forest machine learning methods combined with LLMs to analyze the influencing factors of AKI within 48 h after cardiac surgery, and employed LLMs to simulate the judgment of senior medical heart and kidney experts to improve the medical relevance of feature selection and the clinical interpretability of the model.

## 2 Objects and methods

### 2.1 Data source

The MIMIC-IV database contains multidimensional clinical data of more than 60,000 ICU patients, covering the patients’ basic demographic characteristics, pathological diagnosis, treatment process, laboratory test results, drug use, imaging examinations, vital signs monitoring, and ICU treatment details (6, 7). All data are strictly de-identified to ensure the maximum protection of patient privacy and strictly comply with the privacy protection requirements of the United States Health Insurance Portability and Accountability Act (HIPAA). In this study, the data of patients who underwent cardiac surgery in the ICU were screened, focusing on the occurrence of postoperative AKI and its related risk factors. The included patient data included preoperative and intraoperative clinical indicators (see Table 1).

**TABLE 1:** Study subject selection criteria (8).

Inclusion criteria	Exclusion criteria
Those who meet the AKI-related diagnostic requirements in the “Guidelines for the Management of AKI in the Perioperative Period and ICU”	Kidney transplant recipient
Patient age: 18–80 years	People with cognitive impairment or mental illness
Patient hospital stay > 48 h	Those with kidney-related diseases or medical history
Underwent heart surgery during ICU stay	Family history

The diagnosis of AKI is primarily based on the three stages defined by the Kidney Disease: Improving Global Outcomes (KDIGO) guidelines (see Table 2).

**TABLE 2:** Definition and staging of acute kidney injury (AKI).

Stage	SCr criteria	Urine output criteria
Stage 1	Increase in SCr ≥ 0.3 mg/dL (≥ 26.5 μmol/L), or increase in SCr to ≥ 1.5 times baseline	Urine output < 0.5 mL/kg/h for ≥ 6 h
Stage 2	Increase in SCr to ≥ 2 times baseline	Urine output < 0.5 mL/kg/h for ≥ 12 h
Stage 3	Increase in SCr to ≥ 3 times baseline, or SCr ≥ 4.0 mg/dL (≥ 353.6 μmol/L)	No specific urine output threshold, or initiation of renal replacement therapy (RRT)

### 2.2 Data extraction and processing

Data from the MIMIC-IV database were collected, including age, gender, marital status, and death status; preoperative medication status, including medication time and dosage; and laboratory indicators such as serum creatinine, pulmonary capillary wedge pressure, mean arterial blood pressure, diastolic blood pressure, serum bicarbonate, anion gap, C-reactive protein, hematocrit, vancomycin level, blood oxygen saturation, phosphorus, sodium ion (serum), sodium ion (whole blood), body weight, total arterial carbon dioxide, red blood cells, arterial blood pH, chloride ion (serum), etc., (9, 10). This study extracted patient data within 48 h following cardiac surgery, including both patients who developed AKI and those who did not (11). For patients with AKI, staging was performed according to stages 1, 2, and 3. To better predict the occurrence of postoperative AKI using preoperative clinical indicators, preoperative laboratory test results and medication information were extracted as predictive variables. Missing values for all variables were imputed using multiple imputation methods (12).

This study cleaned the patient information extracted from the MIMIC database. To ensure data quality and consistency, Z-score standardization was used as a data preprocessing method to standardize clinical data from different sources and high dimensions, removing the impact of dimensions and ensuring the balanced contribution of each feature in model training.

### 2.3 Building a random forest machine learning prediction model

In the random forest analysis, the full set of preselected clinical variables—covering both potential confounders and key predictors—was entered into the model without prior exclusion, allowing the algorithm to internally assess their relative importance. Random forests are well-suited for handling continuous (float-type) data, particularly in capturing non-linear relationships and feature interactions. Unlike traditional linear models that rely on the assumption of linearity among features, random forests build multiple decision trees and aggregate their results, enabling the effective identification and modeling of complex patterns within continuous data. By randomly selecting subsets of features and samples during training, random forests reduce the need for strong assumptions or extensive preprocessing of float-type variables, thereby enhancing the model’s adaptability and predictive accuracy (13, 14).

In addition, in terms of feature importance assessment, random forests can quantify the contribution of each feature to the prediction results, help identify key factors closely related to AKI risk, and thus provide strong support for clinical decision-making (15).

### 2.4 Building the LASSO prediction model

The target variable in this study is closely associated with the occurrence of AKI. All candidate predictors, including potential confounders, were initially included simultaneously as multivariable inputs. Subsequently, Lasso regression was applied for variable selection. When dealing with high-dimensional data that contain numerous clinical features, Lasso regression serves as a commonly used feature selection method. By applying L1 regularization, it effectively identifies the features most relevant to the target variable. In datasets with redundant or irrelevant features, Lasso regression automatically shrinks the coefficients of less important variables to zero, thereby reducing model complexity and improving computational efficiency. When applied to numerical data, Lasso regression can effectively assess the importance of continuous variables, demonstrating strong adaptability in high-dimensional settings. Its L1 regularization also helps prevent overfitting, a common issue in high-dimensional data, thus enhancing the model’s generalizability and maintaining robust predictive performance on new data. The predictive features selected through Lasso regression contribute to improved model accuracy. In the context of AKI risk prediction, identifying key variables closely related to AKI onset is of great clinical significance, as it supports early recognition and targeted intervention in clinical practice. In this study, Lasso regression was employed to identify clinical indicators associated with the occurrence of different stages of AKI following cardiac surgery. By optimizing the regularization parameter λ, the model achieves a balance between fitting performance and complexity. The selection of the λ value was optimized through cross-validation to achieve the best fitting effect and control the model complexity. During the training process, the hyperparameters of the Lasso regression model (including λ) were carefully tuned through grid search (GridSearchCV) or other optimization methods to ensure high prediction performance under the optimal configuration.

In addition, to ensure that the label distribution of the training set and the test set is consistent, stratified sampling (stratify = y) is used to ensure that the proportion of each category in both is the same.

### 2.5 Statistical analysis

Continuous variables were summarized as median (interquartile range, IQR) and compared between AKI and non-AKI groups using the Mann–Whitney U test; if normally distributed by the Shapiro–Wilk test, they were reported as mean ± SD and compared with Student’s *t*-test. Categorical variables were presented as *n* (%) and compared using Pearson’s chi-square test (Fisher’s exact test when expected cell counts <5). Two-sided *P*-values <0.05 were considered statistically significant. Where appropriate, *P*-values were adjusted for multiple testing using the Benjamini–Hochberg false discovery rate (FDR) procedure.

To estimate adjusted associations, we fitted multivariable logistic regression models with postoperative AKI (yes/no) as the dependent variable. Candidate predictors included those showing between-group differences in univariate tests and those retained *a priori* for clinical plausibility and LLMs-simulated expert validation. The models were adjusted for potential confounders (age, sex, baseline serum creatinine, and type of cardiac surgery). Linearity in the logit for continuous predictors was assessed (locally weighted smoothed plots); when violated, variables were modeled using restricted cubic splines or clinically meaningful categories. Multicollinearity was evaluated using variance inflation factors (VIF), and predictors with VIF >5 were excluded or combined. Missing data were handled via multiple imputation by chained equations (m = 5); regression estimates were pooled with Rubin’s rules. Because continuous predictors were z-standardized during preprocessing, adjusted odds ratios (ORs) correspond to a 1-SD increase unless otherwise specified. Adjusted ORs with 95% confidence intervals (CIs) were reported and visualized in a forest plot.

### 2.6 LLMs - enhanced AKI risk mechanism analysis

This study used large language models (LLMs) to simulate the thinking process of clinicians and deeply analyzed the relationship between the selected feature variables and the occurrence of AKI. LLMs systematically evaluated the correlation between preoperative indicators and postoperative AKI by deeply analyzing the physiological mechanisms of each variable and integrating clinical medical knowledge. Specifically, LLMs not only analyze the correlation between features through quantitative model, but also explain how these variables affect kidney function and the occurrence of AKI through qualitative reasoning.

When analyzing each characteristic variable, LLMs first analyzed the mechanism of action of the variable in detail from a physiological perspective, combined with known medical knowledge. For example, advanced age, as one of the important factors affecting AKI, may lead to an increased incidence of postoperative AKI by reducing renal reserve function and increasing the risk of complications. Elevated preoperative serum creatinine reflects the impaired state of baseline renal function, suggesting that patients may have a higher risk of renal damage. In addition, the use of vancomycin has been identified as a drug-induced nephrotoxicity mechanism, and a decrease in hematocrit may affect the oxygen supply to the kidneys, further aggravating the occurrence of AKI.

Through positive and negative bidirectional demonstration of the above factors, the study found that the increased preoperative levels of these indicators were significantly positively correlated with the incidence of postoperative AKI (*P* < 0.05). At the same time, the pathways of widening of the anion gap (possibly related to undiagnosed metabolic acidosis) and increased inspired oxygen concentration (FiO_2_) (possibly reflecting the degree of preoperative lung dysfunction) have potential physiological explanations, but due to the lack of sufficient clinical data support, these factors are still labeled as uncertain factors, and more clinical data are needed to further verify their role in the occurrence of AKI.

For example, LLMs pointed out through the analysis of increased serum creatinine that the increase in serum creatinine reflects the state of renal failure, suggesting that patients may be at a higher risk of AKI. In addition, the impact of changes in serum potassium on electrolyte balance and the increased international normalized ratio (INR) that may cause bleeding and hypoperfusion, thereby increasing the risk of AKI, was further analyzed and confirmed by LLMs. In the risk assessment stage, this study combined machine learning algorithms (such as LASSO and random forest) with the results of LLMs analysis to rank the degree of influence of each variable on the occurrence of AKI to better identify and evaluate potential high-risk factors.

In order to improve the reliability of the conclusions, this study used LLMs after memory reset to verify the analysis process multiple times. By resetting the model’s memory, it is ensured that the LLMs’s reasoning process is not disturbed by the previous analysis, thereby further verifying the logical rationality of the original conclusion. The model confirmed the rationality of the analysis process through verification feedback (the feedback was “yes”). In order to enhance the stability and consistency of the results, this study also performed consistency screening through two independent machine learning algorithms (LASSO and random forest) to ensure the consistency of the screened variables in different algorithms, thereby enhancing the reliability of the results.

Large language models not only simulate the decision-making process of doctors, but also help analyze the mechanism of action of each variable. According to the degree of influence of the variable on the occurrence of AKI, LLMs provides corresponding intervention recommendations for each risk factor. Through this simulated decision-making process, LLMs can provide clinicians with more accurate risk assessments, thereby helping doctors make more scientific clinical decisions.

### 2.7 Specific research methods for risk prediction of AKI after cardiac surgery based on large-scale language models (LLMs)

With the rapid advancement of large language models (LLMs), particularly the emergence of cutting-edge models such as GPT-4.5 and Gemini, new research pathways and tools have become available for biomedical data analysis. In this study, we propose a novel AKI risk prediction method for patients undergoing cardiac surgery, based on iterative validation across multiple LLMs. These models include both cloud-based LLMs (ChatGPT-4.5, ChatGPT-4o, Google Gemini 2.5) and locally deployed LLMs (DeepSeek-R1, Gemma 3 27B, Qwen 3 30B), which were used to simulate clinical reasoning by computationally mimicking the decision-making processes of physicians.

To systematically screen and validate predictors, an LLMs-based consensus workflow was applied. Variables with concordant classifications from at least four LLMs were retained, with the majority label assigned as the final risk category, whereas those not meeting this criterion were deemed irrelevant. The resulting consensus list was exported as the final dataset for subsequent model development and interpretability analyses. All online queries in this study were automated through API interfaces, ensuring efficient and scalable data interaction.

#### 2.7.1 Prompt design for biological function analysis

In order to ensure that LLMs can accurately analyze the relationship between changes in key clinical indicators before and during surgery (such as serum creatinine, blood urea nitrogen (BUN), sodium, potassium, etc.) and postoperative AKI, this study designed a precise prompt. The prompt requires LLMs to simulate the role of an expert in AKI risk assessment after cardiac surgery, analyze how changes (increase or decrease) in these clinical indicators affect the occurrence of postoperative AKI, and describe their mechanism of action in biological processes. To ensure the scientificity and objectivity of the analysis, the prompt clearly requires LLMs to remain neutral. If the role of a clinical indicator is unclear, it will be marked as “unclear.” The specific contents of the prompt are as follows:

“Assume that you are an expert in the field of AKI risk analysis after cardiac surgery and are well-versed in the impact of preoperative and intraoperative clinical indicators on the occurrence of postoperative AKI. Please analyze how changes (increase or decrease) in the following clinical indicators during surgery are related to the risk of postoperative AKI, and describe the specific biological processes by which they play a role. If the role of a clinical indicator is unclear, please mark it as “unclear.” The following is a list of clinical indicators: (list of clinical indicators).”

#### 2.7.2 Reducing hallucinations in LLMs output

In order to solve the common illusion phenomenon in LLMs, that is, the model generates inaccurate or inconsistent content, a verification method based on iterative verification and similarity comparison is adopted. This method ensures the reliability and scientificity of each output through multiple verifications and comparisons of results. The specific verification steps are as follows:

First, LLMs generate preliminary output based on the provided prompts, describing the relationship between changes in each clinical indicator and the risk of postoperative AKI. Then, the generated preliminary output is combined with the marker name to form a second input, requiring the model to re-evaluate its effectiveness. The prompts for verification are as follows:

“Please evaluate whether the clinical indicator name and functional description in the input match reasonably. If reasonable, please answer “yes”; if not reasonable, please answer “no” and provide the correct biological role of the clinical indicator, especially its role in the occurrence of postoperative AKI. The specific input is as follows: (clinical indicator name) + initial results.”

#### 2.7.3 Compilation and synthesis of results

**FIGURE 1:**
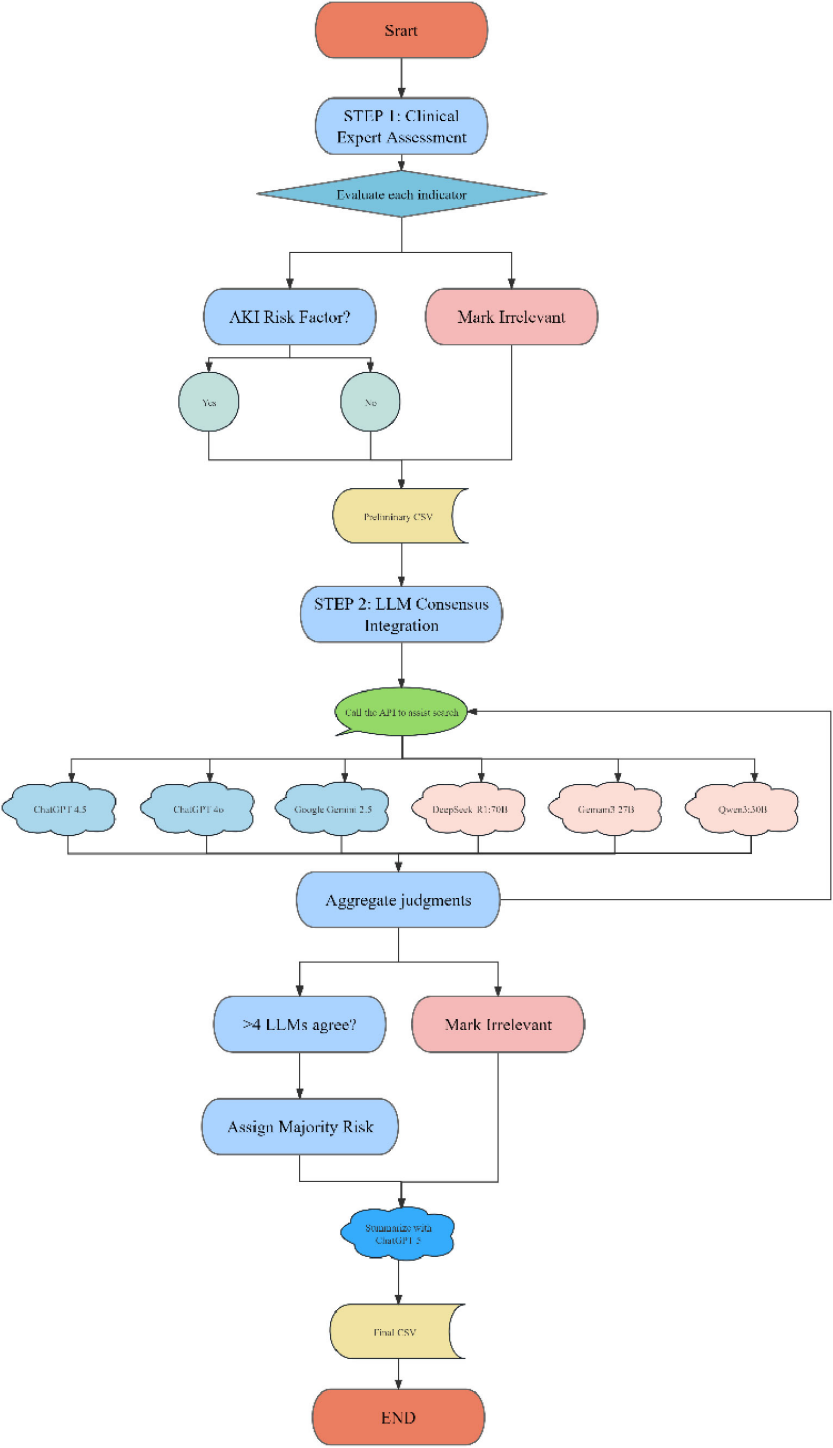
The process of querying the large language models (LLMs).

After multiple verifications and iterations of the LLMs connection API, the final functional description of each clinical indicator was obtained, which clarified the role of each indicator in the occurrence of postoperative AKI. The verified results will be summarized to form a comprehensive assessment of the risk of postoperative AKI. This process ensures that reliable clinical indicator analysis can be used to predict the risk of AKI after cardiac surgery, provide accurate risk assessment, and provide a scientific basis for clinical decision-making (see Figure 1). A detailed workflow is provided in Supplementary materials.

## 3 Results

### 3.1 Patient extraction results and multi-model analysis

**FIGURE 2:**
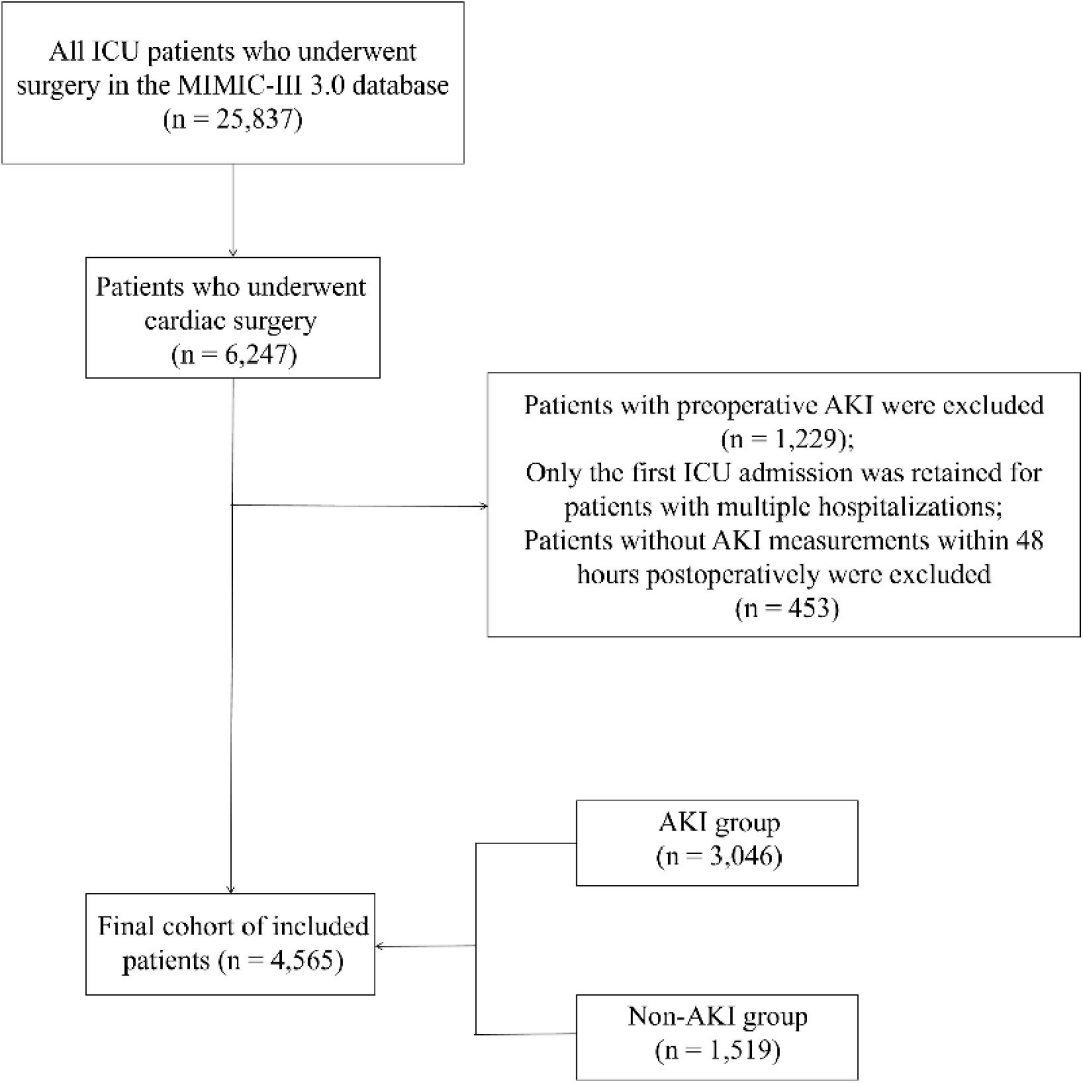
Flowchart of cohort selection from MIMIC-IV.

In the MIMIC-IV database, there are 25,837 ICU patients who underwent surgery, with 6,247 undergoing cardiac surgery. After excluding 1,229 patients with preoperative AKI, the first hospitalization details were retained for patients with repeated admissions. Additionally, 453 patients who did not have AKI measurements within 48 h were excluded. Ultimately, 4,565 patients were included in the study (see Figure 2).

To identify clinical features strongly associated with the occurrence of AKI at different stages (Stage 1, Stage 2, Stage 3), independent predictive models were constructed for each AKI stage. All models were trained using only preoperative clinical variables to evaluate their importance in predicting AKI.

To identify clinical features associated with different stages of AKI (Stage 1, Stage 2, Stage 3), we constructed separate prediction models for each stage. All models were developed using only preoperative clinical variables to ensure real-world clinical applicability and early risk prediction. Feature importance in the random forest models was quantified based on the contribution of each variable to decision tree splits. Higher importance scores indicated stronger predictive relevance. To ensure optimal model performance, the max_features parameter was fine-tuned. To reduce potential confounding, we selected the top-ranking features for each AKI stage and compared them using a Venn diagram to extract shared predictive variables across all three stages. These consistently important variables were considered common predictors of AKI, reflecting their robust predictive power across the clinical spectrum of AKI. Representative features included variables related to fluid therapy, electrolyte management, laboratory markers, medications, and hemodynamic parameters.

In parallel, LASSO regression models were developed separately for each AKI stage. Using L1 regularization, these models effectively reduced high-dimensional feature spaces by shrinking the coefficients of irrelevant or redundant variables toward zero. This approach not only minimized overfitting but also enhanced model generalizability. Notably, as AKI severity increased, the number of significant predictors decreased, suggesting that advanced AKI stages can be predicted with fewer but more decisive variables.

To further determine stage-independent predictors, the top features identified by the LASSO models for each stage were compared using Venn diagram analysis. The intersection revealed a set of core clinical indicators consistently associated with AKI across all stages. These features encompassed various domains, including fluid balance (e.g., Sodium Chloride 0.9% Flush, Potassium Chloride, Free Water), medication use (e.g., Propofol, Atorvastatin, Morphine Sulfate), laboratory results [e.g., Creatinine (serum), BUN, Lactic Acid, Chloride (serum)], and vital signs or respiratory parameters [e.g., Arterial Blood Pressure Mean, PEEP Set, Inspired O2 Fraction, Cardiac Output (thermodilution)]. The integration of these core indicators laid the foundation for robust and interpretable AKI risk prediction models applicable to the perioperative cardiac surgery setting.

### 3.2 Model evaluation

**FIGURE 3:**
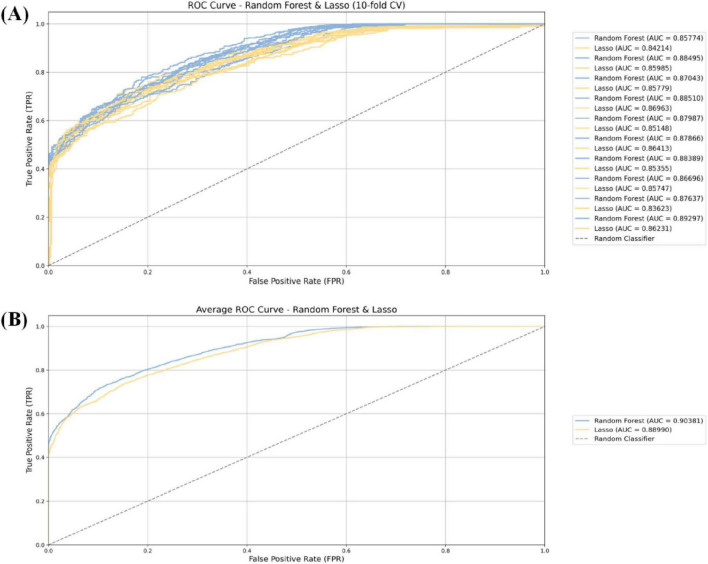
The 10-fold cross-validation method **(A)** and receiver operating characteristic (ROC) curves of various models for predicting postoperative acute kidney injury (AKI) in cardiac **(B)**.

In order to evaluate the predictive performance of multiple machine learning model constructed in this study, the 10-fold cross-validation method was used. In each round of cross-validation, the model was trained using the training set and evaluated on the validation set. Each round of the entire 10-fold cross-validation process outputs the corresponding evaluation indicators, and finally, the overall performance and stability of the model are obtained by averaging all 10-fold cross-validation results. In order to comprehensively measure the ability of the model in predicting the occurrence of AKI, multiple standardized evaluation indicators were calculated, including accuracy, sensitivity, specificity, precision, as well as the receiver operating characteristic curve (ROC) and the area under the ROC curve (AUC). The AUC values are all greater than 0.85, and the model performance has high reliability, strong discrimination ability, and stable predictive performance in the AKI prediction task (see Figure 3 and Table 3).

**TABLE 3:** Model evaluation results.

Model	Accuracy	Sensitivity	Specificity	Precision	AUC (area under the curve)
Random forest	0.76124	0.85247	0.72144	0.72144	0.87769
Lasso regression	0.75781	0.73342	0.77124	0.77124	0.72144

### 3.3 Validation of clinical relevance of predictive factors using large language models (LLMs)

In this study, a variety of advanced large language models (LLMs), including ChatGPT-4.5, ChatGPT-4o, Google Gemini 2.5, DeepSeek-R1, Gemma 3 27B, and Qwen 3 30B, were used to conduct in-depth clinical validation of the predictive factors identified by the LASSO and random forest models. These LLMs, built on extensive medical knowledge and literature, independently evaluated and confirmed the clinical relevance of each predictive factor in relation to postoperative AKI following cardiac surgery.

Through comprehensive analysis, the LLMs consistently indicated that certain predictive factors—such as 0.9% Sodium Chloride, 5% Dextrose, Acetaminophen, Activated Clotting Time, Acyclovir, Amiodarone, Arterial CO_2_ Pressure, Arterial O_2_ Pressure, Atorvastatin, and Dexmedetomidine (Precedex)—though frequently used in clinical practice, lack a clear pathophysiological mechanism or direct association with the development of postoperative AKI. These variables were interpreted as indicators of routine clinical management or general drug administration rather than true causative or predictive factors of renal injury, and thus were excluded from the final model.

To ensure the accuracy of prediction, the LLMs further confirmed 18 key clinical variables as being highly relevant to postoperative AKI. These variables are clinically meaningful and closely related to the biological mechanisms underlying AKI. Among the final predictive factors, age (anchor_age) was widely recognized as a fundamental demographic risk factor. With increasing age, renal structure and function decline, nephron number decreases, and glomerular filtration rate drops, reducing renal reserve capacity and making elderly patients more susceptible to postoperative AKI.

In terms of metabolic and electrolyte indicators, serum creatinine and blood urea nitrogen (BUN) are essential markers of baseline renal function. Elevated levels often indicate pre-existing renal impairment and serve as strong predictors of postoperative AKI. Additionally, serum potassium and serum sodium abnormalities reflect electrolyte disturbances that may disrupt nephron function or contribute to further renal damage. Lactic acid elevation suggests tissue hypoperfusion or hypoxia, making it a sensitive marker of low organ perfusion. Elevated Troponin-T, frequently seen in perioperative myocardial injury, may reduce cardiac output and indirectly impair renal perfusion, thus exacerbating renal dysfunction.

Regarding medication use, Furosemide (Lasix), a commonly used diuretic, can cause hypovolemia or tubular injury if administered excessively, making it an independent risk factor for AKI. Among antibiotics, both Vancomycin (Random) and Gentamicin (Trough) are well-documented nephrotoxic agents, and their use is significantly associated with increased AKI risk. In addition, inappropriate administration of Albumin 5% may indicate imbalances in fluid management strategies, indirectly reflecting a negative impact on renal perfusion.

In the aspect of hemodynamic parameters, reduced arterial blood pressure mean (ART BP Mean) may lead to insufficient glomerular perfusion and is a direct trigger for AKI. Decreased cardiac output (thermodilution) indicates compromised cardiac function, which significantly affects renal perfusion. Brain Natriuretic Peptide (BNP), a sensitive biomarker of volume overload and heart failure, suggests postoperative volume imbalance or cardiac dysfunction and is indirectly associated with AKI development.

For immune response markers, abnormal counts of lymphocytes (Absolute Count - Lymphs), monocytes (Absolute Count - Monos), and neutrophils (Absolute Count - Neuts) are commonly observed in postoperative inflammatory or infectious states. These indicate immune system activation, which plays a critical role in AKI pathogenesis. Persistent inflammation is thought to promote tubular damage and interstitial fibrosis, making changes in immune cell counts important indicators of AKI risk. Additional details are available in Supplementary Table 1.

Through multi-model iterative analysis and verification, the use of LLMs in this study effectively ensured the clinical validity and scientific soundness of selected predictive factors. This validation process not only excluded variables lacking direct clinical significance but also confirmed a core set of features strongly linked to AKI, thereby enhancing the interpretability and reliability of the prediction model. Ultimately, this provides clinicians with a robust foundation for accurate AKI risk assessment, improving prevention and intervention strategies in the postoperative setting.

To further quantify the statistical differences in these clinically validated variables between the AKI and non-AKI groups, chi-square tests were conducted for categorical variables. The analysis showed that most predictors exhibited significant between-group differences (P < 0.05), supporting their strong association with postoperative AKI. Several variables did not reach statistical significance; however, they were retained as postoperative AKI risk factors based on established clinical relevance and confirmation through LLMs-simulated expert validation. Detailed results of the chi-square tests, including the distribution ranges, test statistics, and *p*-values for each variable, are presented in Table 4.

**TABLE 4:** Chi-square test table.

Variable (unit)	All patients	All patients range	Non-AKI group	Non-AKI group range	AKI group	AKI group range	Z/χ^2^ value	*P*-value
anchor_age	67.00	18.00–91.00	66.00	20.00–91.00	68.00	18.00–91.00	30.99	< 0.001
ART BP mean	71.75	32.00–297.00	71.75	42.00–77.00	71.75	32.00–297.00	36.47	< 0.001
Absolute count - Lymphs	1.32	0.00–42.25	1.32	0.00–29.96	1.32	0.00–42.25	4.10	0.043
Absolute count - Monos	0.92	0.00–6.66	0.92	0.00–6.66	0.92	0.00–4.23	112.38	< 0.001
Absolute count - Neuts	12.22	0.02–47.34	12.22	1.22–37.93	12.22	0.02–47.34	139.46	< 0.001
Albumin 5%	0.00	0.00–1000.00	0.00	0.00–500.00	0.00	0.00–1000.00	516.51	< 0.001
Arterial blood pressure mean	72.95	15.00–150.00	72.95	15.00–150.00	72.95	18.00–149.00	2.81	0.093
BUN	37.24	2.00–186.00	37.24	2.00–163.00	37.24	4.00–186.00	90.96	< 0.001
Brain natiuretic peptide (BNP)	12332.69	244.00–68544.00	12332.69	254.00–61696.00	12332.69	244.00–68544.00	4.31	0.038
Cardiac output (thermodilution)	5.11	3.03–8.07	5.11	3.03–5.11	5.11	4.18–8.07	5.48	0.019
Creatinine (serum)	2.29	0.00–15.70	2.29	0.30–11.60	2.29	0.00–15.70	187.51	< 0.001
Furosemide (Lasix)	0.00	0.00–250.00	0.00	0.00–250.00	0.00	0.00–200.00	1097.25	< 0.001
Gentamicin (Trough)	1.48	0.40–3.70	1.48	0.40–2.00	1.48	0.40–3.70	0.19	0.666
Lactic Acid	3.75	0.20–20.00	3.75	0.30–14.00	3.75	0.20–20.00	191.85	< 0.001
Potassium (serum)	4.52	1.50–9.50	4.52	2.20–9.10	4.52	1.50–9.50	84.09	< 0.001
Sodium (serum)	135.11	107.00–167.00	135.11	120.00–161.00	135.11	107.00–167.00	1.16	0.281
Troponin-T	1.40	0.02–24.15	1.40	0.02–20.83	1.40	0.02–24.15	40.19	< 0.001
Vancomycin (Random)	19.76	1.90–43.20	19.76	3.10–41.20	19.76	1.90–43.20	2.95	0.086

**FIGURE 4:**
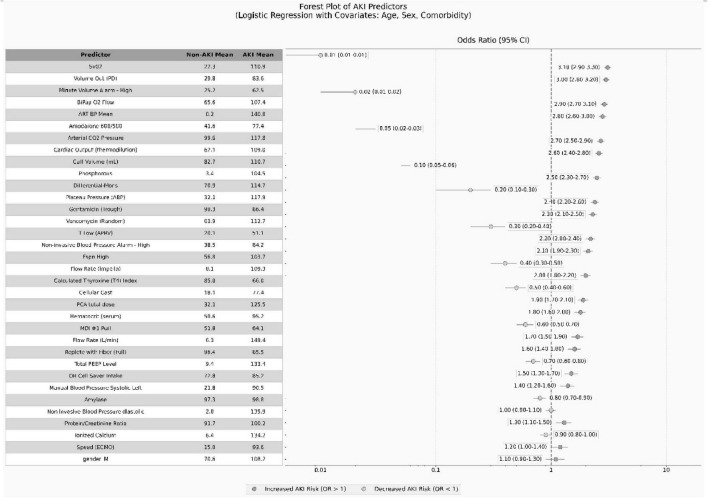
Odds ratios and their confidence intervals for important variables associated with acute kidney injury (AKI).

The figure presents key predictors of AKI through a composite table combining a table and forest plot. The table section displays the means of each variable in the non-AKI and AKI groups, while the forest plot visually illustrates the impact of each variable on AKI risk using confidence intervals and odds ratios (ORs). Horizontal lines and circles represent the confidence intervals and OR values for each variable, with ORs > 1 marked by blue circles (indicating increased risk) and ORs < 1 marked by green circles (indicating reduced risk). Text labels directly display the OR values and their confidence interval ranges. The figure legend explicitly states the use of a logistic regression model and mentions covariates including age, sex, and comorbidities. A vertical reference line (OR = 1) is added to the right chart, along with a color-coded legend indicating the direction of risk, enabling clear presentation of AKI predictors and facilitating direct comparison of differences between groups (see Figure 4).

### 3.4 Construction of the AKI risk-prediction model

By constructing an AKI risk prediction model (AKI RISK ASSESSMENT), the data was divided into a test set (30%) and a training set (70%). The model accuracy was as high as 72.87%, where the coefficients of each feature included the coefficient values of each feature [such as Anion gap, Creatinine (serum), etc.,], which represent the impact of each feature on the target variable (illness) (see Table 5).


$$A\,R\,A=\underset{i=1}{\sum }x_{i}\times w\,e\,i\,g\,h\,t_{i}$$

**TABLE 5:** Prediction model feature values and their parameters.

X	Feature	Weight
X_1_	anchor_age (basic information)	0.07809
X_2_	Creatinine (serum) (metabolism and electrolytes)	0.26451
X_3_	Furosemide (Lasix) (medication)	−0.36554
X_4_	Gentamicin (Trough) (medication)	0.02833
X_5_	Albumin 5% (medication)	−0.01347
X_6_	Troponin-T (metabolism amd electrolytes)	0.00768
X_7_	Vancomycin (Random) (medication)	0.01271
X_8_	BUN (blood urea nitrogen) (metabolism and electrolytes)	−0.04189
X_9_	Potassium (serum) (metabolism and electrolytes)	0.29116
X_10_	Sodium (serum) (metabolism and electrolytes)	0.02379
X_11_	Lactic acid (metabolism and electrolytes)	0.041416
X_12_	ART BP mean (hemodynamics)	−0.05194
X_13_	Arterial blood pressure mean (hemodynamics)	−0.00516
X_14_	Cardiac output (thermodilution) (hemodynamics)	0.00484
X_15_	Brain natriuretic peptide (BNP) (hemodynamics)	−0.01135
X_16_	Absolute count - Lymphs (immune response)	−0.00686
X_17_	Absolute count - Monos (immune response)	−0.01507
X_18_	Absolute count - Neuts (immune response)	−0.01569

Formula 1: Aki risk assessment.

### 3.5 Model interpretability and feature visualization analysis

To improve the transparency, clinical interpretability, and decision support utility of the prediction model, we conducted comprehensive model interpretability and feature visualization analyses. These included feature correlation analysis, SHAP value interpretation, and multivariate correlation heatmaps.

**FIGURE 5:**
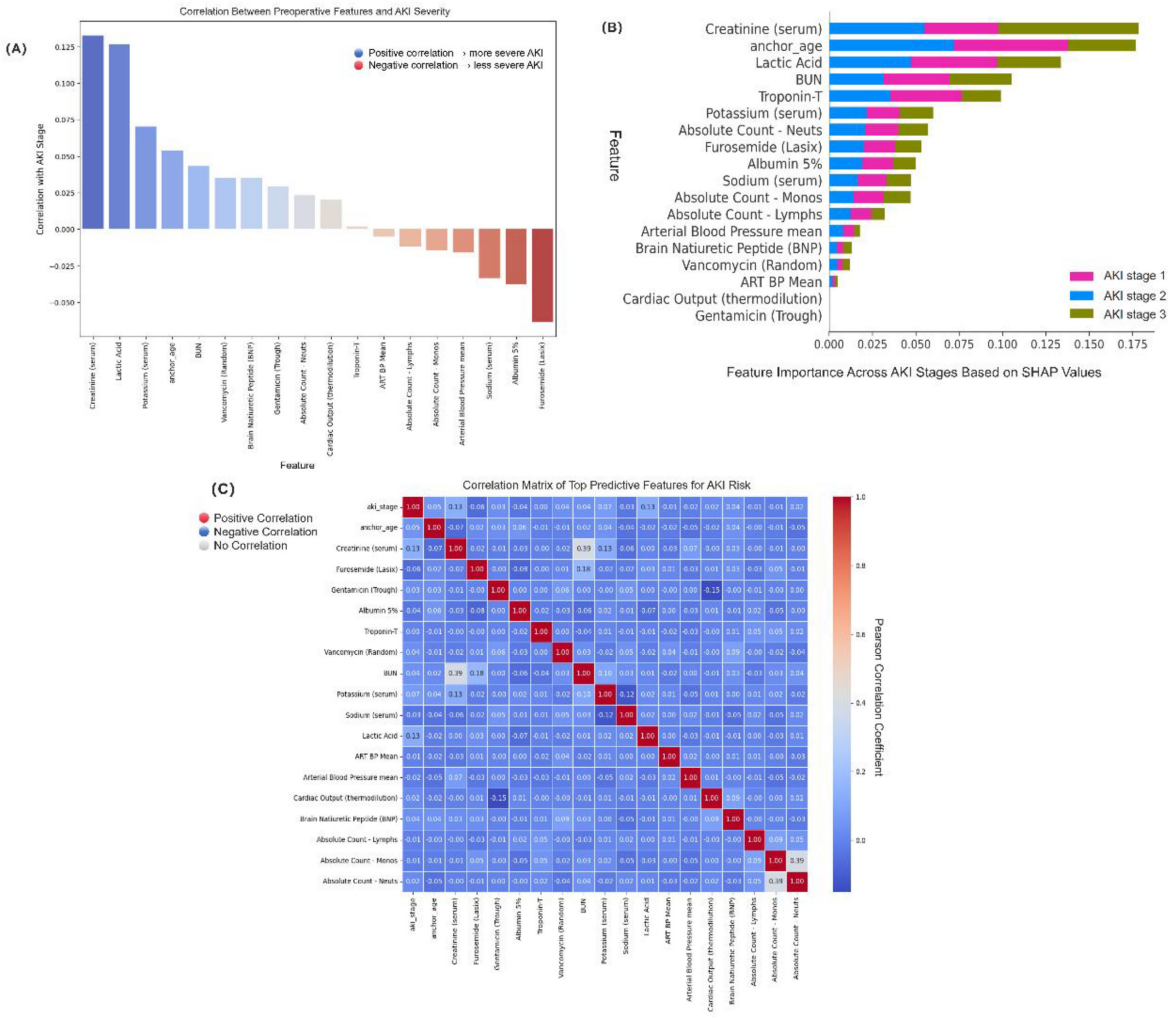
Model interpretability and key feature visualization. **(A)** Correlation between each feature and acute kidney injury (AKI) stage (Stage 0–3). Positive values (blue) indicate association with more severe AKI, while negative values (red) indicate a protective or inverse relationship. **(B)** SHapley Additive exPlanations (SHAP) summary plot showing the average impact of top features on model predictions for each AKI stage. The color-coded bars represent AKI stage 1 (pink), stage 2 (blue), and stage 3 (olive green). **(C)** Pearson correlation heatmap among top predictive features. Red indicates strong positive correlation, blue indicates strong negative correlation, and white indicates no correlation. The color bar reflects the Pearson correlation coefficient ranging from −1 to +1.3.

Feature correlation analysis demonstrated the strength and direction of linear associations between each variable and the AKI stage (Figure 5A). Creatinine (serum), lactic acid, and potassium (serum) showed the strongest positive correlations with AKI severity, suggesting their important roles in reflecting renal dysfunction and tissue hypoperfusion. Conversely, features such as furosemide (Lasix), albumin 5%, and sodium (serum) were negatively correlated with AKI stage, potentially indicating their association with volume management and treatment interventions.

To explore potential multicollinearity and inter-feature relationships, we constructed a correlation heatmap using Pearson’s correlation coefficient (Figure 5B). While most variables demonstrated weak-to-moderate intercorrelations, we observed a notable positive correlation between serum creatinine and BUN (r = 0.39), as well as between absolute neutrophil and monocyte counts (r = 0.39), consistent with their shared physiological roles in renal function and immune response. Cardiac Output (thermodilution) and Gentamicin (Trough) do not display visible bars because their mean absolute SHAP values were near zero at the plotting scale for some AKI stages. Both predictors were nevertheless retained based on external clinical validation (LLMs consensus plus nephrologist adjudication) and strong mechanistic plausibility—reduced cardiac output reflects renal hypoperfusion, while aminoglycosides (gentamicin) have well-described nephrotoxicity.

For individualized prediction interpretability, we applied SHAP (SHapley Additive exPlanations) analysis, which estimates the marginal contribution of each feature to the model’s output for each AKI stage class (Figure 5C). The SHAP summary plot revealed that furosemide (Lasix), anchor_age, albumin 5%, lactic acid, and creatinine (serum) had the highest average impact across AKI severity classes (AKI stage 1–3), indicating these features play pivotal roles in AKI risk discrimination. Importantly, while anchor_age and creatinine were major contributors in all classes, some features such as BNP and neutrophil count showed stage-specific influence, highlighting the heterogeneous pathophysiology of AKI progression.

Collectively, the interpretability framework confirmed the clinical relevance and robustness of the selected features and provided clinicians with a transparent basis for understanding the model’s prediction process. This visualization strategy not only improved the model’s transparency but also reinforced the trustworthiness of AI-assisted decision-making in perioperative AKI risk management.

## 4 Discussion

This study focused on the risk prediction of AKI following cardiac surgery. Based on the MIMIC-IV database, machine learning models were constructed using LASSO regression and random forest algorithms. For the first time, multiple large language models (LLMs) were integrated to validate the clinical relevance of the selected predictive variables. The results demonstrated that this approach not only improved the scientific rigor and rationality of feature selection but also enhanced the clinical interpretability and practical applicability of the predictive models.

While conventional statistical and machine learning methods perform well in variable selection, they often rely solely on statistical correlations between variables and outcomes, making it difficult to determine whether a variable holds true clinical significance. To address this limitation, we utilized six mainstream LLMs—ChatGPT-4.5, ChatGPT-4o, Google Gemini 2.5, DeepSeek-R1, Gemma 3 27B, and Qwen 3 30B—to analyze and reason through each initially selected variable. Ultimately, 18 key predictors were confirmed to be clinically meaningful, supported by clear pathophysiological mechanisms. Meanwhile, a number of statistically significant but clinically irrelevant features—such as intravenous fluid infusions, electrolyte supplement medications, and certain sedative or analgesic agents—were excluded from the final model.

The final set of predictors encompassed a wide range of clinically relevant domains, including basic demographic information [e.g., age (16)], metabolic and electrolyte markers [e.g., serum creatinine (17), BUN, lactate, and electrolytes], hemodynamic parameters (e.g., mean arterial pressure, cardiac output, BNP), immune-inflammatory indicators (e.g., absolute neutrophil and monocyte counts), and perioperative medications with known nephrotoxic potential [e.g., furosemide, vancomycin (18), and gentamicin]. These features reflect the multifactorial and multi-pathway pathogenesis of AKI. Not only were they statistically robust, but they are also well-supported by current medical literature and clinical guidelines, achieving a solid balance between theoretical depth and real-world applicability in the proposed prediction model.

In terms of model evaluation, both random forest and LASSO model showed high predictive ability, especially in terms of sensitivity and specificity. Compared with traditional statistical methods, machine learning models can fully explore the complex non-linear relationships in high-dimensional data, which makes AKI risk prediction more accurate and powerful. Compared with previous linear regression model, this study effectively improved the stability and reliability of the model through multi-stage feature screening by adopting Lasso regression, an election mechanism, and 10-fold cross-validation. This method successfully identified the key clinical features of AKI after cardiac surgery and ensured the scientificity and applicability of these features in clinical practice, further enhancing the theoretical basis for individualized AKI risk prediction.

In addition, the application of LLMs in this study simulated the judgment process of senior medical experts and further verified the clinical relevance of feature selection. By simulating real doctors to analyze feature quantities through LLMs, the accuracy and reliability of the selected features were ensured, thereby improving the clinical interpretability of the model. It is worth noting that when LLMs conduct in-depth analysis of clinical data, they can simulate the clinical decision-making process of experts, identify potential risk factors, and provide targeted intervention recommendations for clinicians. This method provides new ideas for the application of artificial intelligence in the medical field and demonstrates the potential of LLMs in improving medical research.

Although this study demonstrated the application prospects of machine learning model in AKI risk prediction, there are still some limitations. First, the data of the MIMIC-IV database mainly comes from a single medical institution, so the generalization ability of the model may be limited to a certain extent. In order to improve the universality of the model, future studies should consider using data from multiple centers for verification. Second, although this study has screened and evaluated features through multiple methods, future studies can combine more real-time monitoring data, such as physiological parameters and drug usage, to further improve the real-time prediction ability of the model. In addition, this study mainly relies on static clinical data, and the dynamic changes of patients in the clinical environment are an important factor in the occurrence of AKI. Therefore, combining dynamic data and time series analysis methods may help improve the accuracy of the model.

In summary, the AKI risk prediction model constructed in this study, based on the MIMIC-IV database and LLMs technology, performed well in terms of accuracy, sensitivity, and clinical interpretability, providing a scientific basis for the early identification and clinical intervention of AKI after cardiac surgery. Through in-depth analysis of clinical data by machine learning, this study not only identified multiple key predictors but also provided theoretical support for constructing individualized risk-prediction models. With the continuous development of technology, this model is expected to be widely used in clinical practice in the future, providing new tools for the prevention and treatment of AKI and promoting the in-depth application of artificial intelligence in the medical field.

## Data Availability

The original contributions presented in this study are included in this article/Supplementary material, further inquiries can be directed to the corresponding authors.
