# Supplementary material for: Integration of machine learning and large language models for screening and identifying key risk factors of acute kidney injury after cardiac surgery
Source: Front Med (Lausanne). 2025 Nov 6;12:1618222. doi: 10.3389/fmed.2025.1618222 (PMC12631415; doi:10.3389/fmed.2025.1618222)
Supplement: Supplementary file 1 [file Data_Sheet_1.docx]

**Supplemental materials**

**1. Why Use Large Language Models (LLMs)**

With the rapid development of large language models (LLMs), particularly advanced models like GPT-4.5 and Gemini, new research pathways and tools have emerged in the field of biomedical data analysis. This study proposes a novel method for predicting acute kidney injury (AKI) risk in cardiac surgery patients, leveraging multiple large language models for iterative validation. These models include cloud-deployed ChatGPT-4.5, ChatGPT-4o, Google Gemini 2.5, as well as locally deployed models like DeepSeek-R1:70B, Gemma 3 27B, and Qwen 3 30B. By simulating clinical decision-making processes, these models allow for the simulation of clinical reasoning.

To systematically screen and validate predictive factors, we employed a large-scale consensus analysis workflow based on language models. Variables that received consistent classification results from at least four LLMs were retained, with the final risk category determined by the majority label. Variables not meeting this criterion were considered irrelevant. The consensus list derived from this analysis was exported as the final dataset for subsequent model development and interpretability analysis. All online queries in this study were automatically processed through API interfaces, ensuring efficient and scalable data interactions.

**(1) Rapid Design of Biomedical Function Analysis**

To ensure that large language models accurately analyze the relationship between key clinical indicators (such as serum creatinine, blood urea nitrogen (BUN), sodium, potassium, etc.) and postoperative AKI risk, we designed a precise questioning scheme. This scheme required the language models to simulate the role of an AKI risk assessment expert following cardiac surgery, analyzing how the changes in these clinical indicators (whether increased or decreased) influence the occurrence of postoperative AKI, and elucidating their biological mechanisms. To ensure the scientific and objective nature of the analysis, the scheme explicitly required the models to maintain a neutral stance. If the effect of a clinical indicator was unclear, it was marked as "uncertain." The specific questioning content was as follows:

"Assume you are an expert in the field of postoperative AKI risk analysis. You have in-depth research on the impact of preoperative and intraoperative clinical indicators on postoperative AKI. Please analyze the correlation between changes (increased or decreased) in the following clinical indicators during the surgery and the risk of postoperative AKI, and explain their biological mechanisms. If the role of any clinical indicator is unclear, please mark it as 'uncertain.' The following is the list of clinical indicators: [List of clinical indicators]."

**(2) Reducing Hallucination in LLM Outputs**

To address the common phenomenon of hallucination in LLMs, where models generate inaccurate or inconsistent content, we employed a validation method based on iterative verification and similarity comparison. This method ensured the reliability and scientific validity of each output through multiple rounds of validation and result comparison. The specific verification steps were as follows:

First, the LLM generated an initial output based on the provided prompt, describing the relationship between changes in clinical indicators and the risk of postoperative AKI. Next, the generated preliminary output was combined with the labeled names to form a second input, requiring the model to re-evaluate its validity. The validation prompt was as follows:

"Please assess whether the clinical indicator names and their functional descriptions are reasonable. If they are, please respond 'Yes.' If not, please respond 'No' and provide the correct biological function of the clinical indicator, particularly its role in the occurrence of postoperative AKI. The specific input is as follows: [Clinical indicator name] + initial result."

**(3) Compiling and Integrating Results**

After multiple rounds of validation and iterative testing through the LLM API connections, we ultimately clarified the functional descriptions of each clinical indicator, clearly revealing their roles in the development of postoperative AKI. Using ChatGPT 5 to integrate the verification results, we constructed a comprehensive risk assessment system for postoperative AKI. This process ensured that the analysis, based on reliable clinical indicators, could effectively predict AKI risk following cardiac surgery, providing scientific evidence for precise risk assessment and establishing a scientific basis for clinical decision-making (see Figure S1).


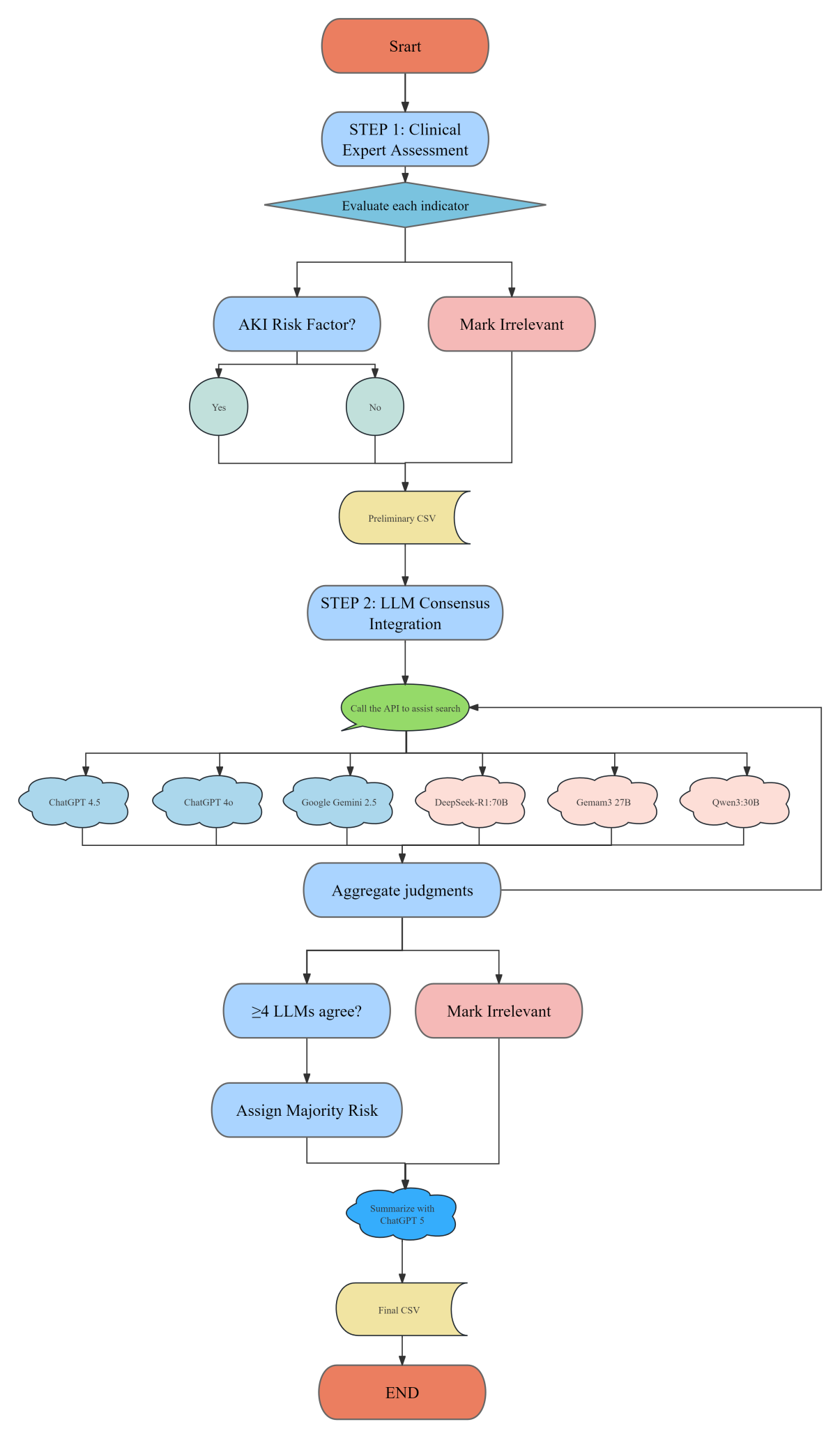


Figure S1: Flowchart of AKI-Assisted Analysis Process

Table S1: Final Exported AKI Results

| Indicator Name | Is AKI Risk Factor | Explanation |
| --- | --- | --- |
| 0.9% Sodium Chloride | No | 0.9% Sodium Chloride is not a primary factor for AKI based on current evidence. |
| 5% Dextrose | No | 5% Dextrose is not a primary factor for AKI based on current evidence. |
| ALT | Yes | ALT is a known clinical marker for AKI. |
| ART BP Mean | Yes | ART BP Mean is a known clinical marker for AKI. |
| AST | Yes | AST is a known clinical marker for AKI. |
| Absolute Count - Lymphs | Yes | Absolute Count - Lymphs is a known clinical marker for AKI. |
| Absolute Count - Monos | Yes | Absolute Count - Monos is a known clinical marker for AKI. |
| Absolute Count - Neuts | Yes | Absolute Count - Neuts is a known clinical marker for AKI. |
| Absolute Eosinophil Count | No | Absolute Eosinophil Count is not a primary factor for AKI based on current evidence. |
| Absolute Lymphocyte Count | Yes | Absolute Lymphocyte Count is a known clinical marker for AKI. |
| Absolute Monocyte Count | Yes | Absolute Monocyte Count is a known clinical marker for AKI. |
| Absolute Neutrophil Count | Yes | Absolute Neutrophil Count is a known clinical marker for AKI. |
| Acetaminophen-IV | No | Acetaminophen-IV is not a primary factor for AKI based on current evidence. |
| Acetaminophen_x | No | Acetaminophen_x is not a primary factor for AKI based on current evidence. |
| Activated Clotting Time | No | Activated Clotting Time is not a primary factor for AKI based on current evidence. |
| Acyclovir | No | Acyclovir is not a primary factor for AKI based on current evidence. |
| Albumin 5% | Yes | Albumin 5% is a known clinical marker for AKI. |
| Alkaline Phosphate | No | Alkaline Phosphate is not a primary factor for AKI based on current evidence. |
| Amiodarone | No | Amiodarone is not a primary factor for AKI based on current evidence. |
| Amiodarone 450/250 | Yes | Amiodarone 450/250 is a known clinical marker for AKI. |
| Amiodarone 600/500 | Yes | Amiodarone 600/500 is a known clinical marker for AKI. |
| Amylase | No | Amylase is not a primary factor for AKI based on current evidence. |
| Anion gap | No | Anion gap is not a primary factor for AKI based on current evidence. |
| Arterial Blood Pressure mean | Yes | Arterial Blood Pressure mean is a known clinical marker for AKI. |
| Arterial CO2 Pressure | No | Arterial CO2 Pressure is not a primary factor for AKI based on current evidence. |
| Arterial O2 pressure | No | Arterial O2 pressure is not a primary factor for AKI based on current evidence. |
| Atorvastatin | No | Atorvastatin is not a primary factor for AKI based on current evidence. |
| BIS Index Range | No | BIS Index Range is not a primary factor for AKI based on current evidence. |
| BUN | Yes | BUN is a known clinical marker for AKI. |
| Bag | No | Bag is not a primary factor for AKI based on current evidence. |
| BiPap O2 Flow | No | BiPap O2 Flow is not a primary factor for AKI based on current evidence. |
| Brain Natiuretic Peptide (BNP) | Yes | Brain Natiuretic Peptide (BNP) is a known clinical marker for AKI. |
| CK (CPK) | Yes | CK (CPK) is a known clinical marker for AKI. |
| CK-MB | Yes | CK-MB is a known clinical marker for AKI. |
| CK-MB Index | Yes | CK-MB Index is a known clinical marker for AKI. |
| CK-MB fraction (%) | Yes | CK-MB fraction (%) is a known clinical marker for AKI. |
| Calcium Gluconate | Yes | Calcium Gluconate is a known clinical marker for AKI. |
| Calcium non-ionized | Yes | Calcium non-ionized is a known clinical marker for AKI. |
| Calculated Thyroxine (T4) Index | Yes | Calculated Thyroxine (T4) Index is a known clinical marker for AKI. |
| Cardiac Output (thermodilution) | Yes | Cardiac Output (thermodilution) is a known clinical marker for AKI. |
| Cath Lab Intake | Yes | Cath Lab Intake is a known clinical marker for AKI. |
| Cellular Cast | Yes | Cellular Cast is a known clinical marker for AKI. |
| Chloride (serum) | Yes | Chloride (serum) is a known clinical marker for AKI. |
| Chloride (whole blood) | Yes | Chloride (whole blood) is a known clinical marker for AKI. |
| Creatinine (serum) | Yes | Creatinine (serum) is a known clinical marker for AKI. |
| D5 1/2NS | Yes | D5 1/2NS is a known clinical marker for AKI. |
| D5W | Yes | D5W is a known clinical marker for AKI. |
| Dextrose 5% | Yes | Dextrose 5% is a known clinical marker for AKI. |
| Differential - Immature Granulocytes | Yes | Differential - Immature Granulocytes is a known clinical marker for AKI. |
| Differential-Eos | Yes | Differential-Eos is a known clinical marker for AKI. |
| Differential-Lymphs | Yes | Differential-Lymphs is a known clinical marker for AKI. |
| Differential-Monos | Yes | Differential-Monos is a known clinical marker for AKI. |
| Differential-Neuts | Yes | Differential-Neuts is a known clinical marker for AKI. |
| Diltiazem | Yes | Diltiazem is a known clinical marker for AKI. |
| Dopamine | Yes | Dopamine is a known clinical marker for AKI. |
| Eosinophils | Yes | Eosinophils is a known clinical marker for AKI. |
| Epinephrine | Yes | Epinephrine is a known clinical marker for AKI. |
| Fentanyl (Concentrate) | Yes | Fentanyl (Concentrate) is a known clinical marker for AKI. |
| Fentanyl_x | Yes | Fentanyl_x is a known clinical marker for AKI. |
| Fibrinogen | Yes | Fibrinogen is a known clinical marker for AKI. |
| Flow Rate (Impella) | Yes | Flow Rate (Impella) is a known clinical marker for AKI. |
| Flow Rate (L/min) | Yes | Flow Rate (L/min) is a known clinical marker for AKI. |
| Free Water | Yes | Free Water is a known clinical marker for AKI. |
| Fresh Frozen Plasma | Yes | Fresh Frozen Plasma is a known clinical marker for AKI. |
| Furosemide (Lasix) | Yes | Furosemide (Lasix) is a known clinical marker for AKI. |
| Furosemide (Lasix) 250/50 | Yes | Furosemide (Lasix) 250/50 is a known clinical marker for AKI. |
| GT Flush | No | GT Flush is not a primary factor for AKI based on current evidence. |
| Gastric Meds | No | Gastric Meds is not a primary factor for AKI based on current evidence. |
| Gentamicin (Trough) | Yes | Gentamicin (Trough) is a known clinical marker for AKI. |
| Glucagon | No | Glucagon is not a primary factor for AKI based on current evidence. |
| Glucose (serum) | Yes | Glucose (serum) is a known clinical marker for AKI. |
| Glucose (whole blood) | Yes | Glucose (whole blood) is a known clinical marker for AKI. |
| Glucose finger stick (range 70-100) | Yes | Glucose finger stick (range 70-100) is a known clinical marker for AKI. |
| HCO3 (serum) | No | HCO3 (serum) is not a primary factor for AKI based on current evidence. |
| Heart Rate | No | Heart Rate is not a primary factor for AKI based on current evidence. |
| Hematocrit (serum) | No | Hematocrit (serum) is not a primary factor for AKI based on current evidence. |
| Heparin Sodium | No | Heparin Sodium is not a primary factor for AKI based on current evidence. |
| Heparin Sodium (Prophylaxis) | Yes | Heparin Sodium (Prophylaxis) is a known clinical marker for AKI. |
| Heparin_x | Yes | Heparin_x is a known clinical marker for AKI. |
| Hydromorphone (Dilaudid) | Yes | Hydromorphone (Dilaudid) is a known clinical marker for AKI. |
| INR | Yes | INR is a known clinical marker for AKI. |
| Immunoglobulin A | No | Immunoglobulin A is not a primary factor for AKI based on current evidence. |
| Inspiratory Time | No | Inspiratory Time is not a primary factor for AKI based on current evidence. |
| Inspired O2 Fraction | Yes | Inspired O2 Fraction is a known clinical marker for AKI. |
| Insulin - Glargine | Yes | Insulin - Glargine is a known clinical marker for AKI. |
| Insulin - Humalog | Yes | Insulin - Humalog is a known clinical marker for AKI. |
| Insulin - Regular | Yes | Insulin - Regular is a known clinical marker for AKI. |
| Ionized Calcium | No | Ionized Calcium is not a primary factor for AKI based on current evidence. |
| KCL (Bolus) | No | KCL (Bolus) is not a primary factor for AKI based on current evidence. |
| LDH | No | LDH is not a primary factor for AKI based on current evidence. |
| LR | Yes | LR is a known clinical marker for AKI. |
| Labetalol | Yes | Labetalol is a known clinical marker for AKI. |
| Lactated Ringers | No | Lactated Ringers is not a primary factor for AKI based on current evidence. |
| Lactic Acid | Yes | Lactic Acid is a known clinical marker for AKI. |
| Lidocaine | No | Lidocaine is not a primary factor for AKI based on current evidence. |
| Magnesium Sulfate | No | Magnesium Sulfate is not a primary factor for AKI based on current evidence. |
| Magnesium Sulfate (Bolus) | No | Magnesium Sulfate (Bolus) is not a primary factor for AKI based on current evidence. |
| Manual Blood Pressure Systolic Left | No | Manual Blood Pressure Systolic Left is not a primary factor for AKI based on current evidence. |
| Meropenem | No | Meropenem is not a primary factor for AKI based on current evidence. |
| Metoprolol | No | Metoprolol is not a primary factor for AKI based on current evidence. |
| Midazolam (Versed) | No | Midazolam (Versed) is not a primary factor for AKI based on current evidence. |
| Milrinone | Yes | Milrinone is a known clinical marker for AKI. |
| Minute Volume Alarm - High | No | Minute Volume Alarm - High is not a primary factor for AKI based on current evidence. |
| Morphine Sulfate | No | Morphine Sulfate is not a primary factor for AKI based on current evidence. |
| NaCl 0.45% | No | NaCl 0.45% is not a primary factor for AKI based on current evidence. |
| NaCl 0.9% | Yes | NaCl 0.9% is a known clinical marker for AKI. |
| Nicardipine | Yes | Nicardipine is a known clinical marker for AKI. |
| Nitroglycerin | Yes | Nitroglycerin is a known clinical marker for AKI. |
| Nitroprusside | Yes | Nitroprusside is a known clinical marker for AKI. |
| Non Invasive Blood Pressure diastolic | Yes | Non Invasive Blood Pressure diastolic is a known clinical marker for AKI. |
| Non-Invasive Blood Pressure Alarm - High | Yes | Non-Invasive Blood Pressure Alarm - High is a known clinical marker for AKI. |
| Norepinephrine | Yes | Norepinephrine is a known clinical marker for AKI. |
| Nucleated Red Cells | Yes | Nucleated Red Cells is a known clinical marker for AKI. |
| O2 Flow | Yes | O2 Flow is a known clinical marker for AKI. |
| O2 saturation pulseoxymetry | No | O2 saturation pulseoxymetry is not a primary factor for AKI based on current evidence. |
| OR Cell Saver Intake | No | OR Cell Saver Intake is not a primary factor for AKI based on current evidence. |
| OR Crystalloid Intake | Yes | OR Crystalloid Intake is a known clinical marker for AKI. |
| OR FFP Intake | Yes | OR FFP Intake is a known clinical marker for AKI. |
| OR Packed RBC Intake | Yes | OR Packed RBC Intake is a known clinical marker for AKI. |
| OR Platelet Intake | Yes | OR Platelet Intake is a known clinical marker for AKI. |
| P High (APRV) | Yes | P High (APRV) is a known clinical marker for AKI. |
| PCA total dose | Yes | PCA total dose is a known clinical marker for AKI. |
| PCWP | Yes | PCWP is a known clinical marker for AKI. |
| PEEP set | Yes | PEEP set is a known clinical marker for AKI. |
| PH (Arterial) | Yes | PH (Arterial) is a known clinical marker for AKI. |
| PH (Venous) | Yes | PH (Venous) is a known clinical marker for AKI. |
| PO Intake | Yes | PO Intake is a known clinical marker for AKI. |
| PO2 (Mixed Venous) | Yes | PO2 (Mixed Venous) is a known clinical marker for AKI. |
| PSV Level | Yes | PSV Level is a known clinical marker for AKI. |
| Packed Red Blood Cells | Yes | Packed Red Blood Cells is a known clinical marker for AKI. |
| Phenylephrine | No | Phenylephrine is not a primary factor for AKI based on current evidence. |
| Phosphorous | No | Phosphorous is not a primary factor for AKI based on current evidence. |
| Piggyback | No | Piggyback is not a primary factor for AKI based on current evidence. |
| Plateau Pressure (IABP) | No | Plateau Pressure (IABP) is not a primary factor for AKI based on current evidence. |
| Potassium (serum) | Yes | Potassium (serum) is a known clinical marker for AKI. |
| Potassium (whole blood) | Yes | Potassium (whole blood) is a known clinical marker for AKI. |
| Potassium Chloride | Yes | Potassium Chloride is a known clinical marker for AKI. |
| Pre-Admission/Non-ICU Intake | Yes | Pre-Admission/Non-ICU Intake is a known clinical marker for AKI. |
| Propofol | No | Propofol is not a primary factor for AKI based on current evidence. |
| Protein/Creatinine Ratio | No | Protein/Creatinine Ratio is not a primary factor for AKI based on current evidence. |
| Prothrombin time | No | Prothrombin time is not a primary factor for AKI based on current evidence. |
| QTc | Yes | QTc is a known clinical marker for AKI. |
| RDW | Yes | RDW is a known clinical marker for AKI. |
| RDW-SD | Yes | RDW-SD is a known clinical marker for AKI. |
| Red Blood Cells | Yes | Red Blood Cells is a known clinical marker for AKI. |
| Respiratory Rate (Set) | Yes | Respiratory Rate (Set) is a known clinical marker for AKI. |
| Reticulocyte Count, Automated | No | Reticulocyte Count, Automated is not a primary factor for AKI based on current evidence. |
| Senna | No | Senna is not a primary factor for AKI based on current evidence. |
| Sodium (serum) | Yes | Sodium (serum) is a known clinical marker for AKI. |
| Sodium (whole blood) | Yes | Sodium (whole blood) is a known clinical marker for AKI. |
| Sodium Bicarbonate 8.4% | No | Sodium Bicarbonate 8.4% is not a primary factor for AKI based on current evidence. |
| Sodium Chloride 0.9% Flush | Yes | Sodium Chloride 0.9% Flush is a known clinical marker for AKI. |
| Solution | Yes | Solution is a known clinical marker for AKI. |
| Speed (ECMO) | Yes | Speed (ECMO) is a known clinical marker for AKI. |
| Sterile Water | No | Sterile Water is not a primary factor for AKI based on current evidence. |
| SvO2 | Yes | SvO2 is a known clinical marker for AKI. |
| T Low (APRV) | No | T Low (APRV) is not a primary factor for AKI based on current evidence. |
| TCO2 (calc) Arterial | No | TCO2 (calc) Arterial is not a primary factor for AKI based on current evidence. |
| Temperature Fahrenheit | No | Temperature Fahrenheit is not a primary factor for AKI based on current evidence. |
| Temporary Atrial Stim Threshold mA | Yes | Temporary Atrial Stim Threshold mA is a known clinical marker for AKI. |
| Thyroid Stimulating Hormone | No | Thyroid Stimulating Hormone is not a primary factor for AKI based on current evidence. |
| Tidal Volume (set) | No | Tidal Volume (set) is not a primary factor for AKI based on current evidence. |
| Total Bilirubin | Yes | Total Bilirubin is a known clinical marker for AKI. |
| Total PEEP Level | Yes | Total PEEP Level is a known clinical marker for AKI. |
| Troponin-T | Yes | Troponin-T is a known clinical marker for AKI. |
| Vancomycin (Random) | Yes | Vancomycin (Random) is a known clinical marker for AKI. |
| Vancomycin_x | Yes | Vancomycin_x is a known clinical marker for AKI. |
| Vasopressin | Yes | Vasopressin is a known clinical marker for AKI. |
| Venous CO2 Pressure | No | Venous CO2 Pressure is not a primary factor for AKI based on current evidence. |
| Venous O2 Pressure | Yes | Venous O2 Pressure is a known clinical marker for AKI. |
| Volume Out (PD) | Yes | Volume Out (PD) is a known clinical marker for AKI. |
| anchor_age | Yes | anchor_age is a known clinical marker for AKI. |
| Indicator Name | Is AKI Risk Factor | Explanation |
| 0.9% Sodium Chloride | No | 0.9% Sodium Chloride is not a primary factor for AKI based on current evidence. |

**2. Example of Using LLMs for AKI Search**

**(1)Here's an example of Creatinine (serum) asking LLMs：**

Assume you are an expert in the field of postoperative AKI risk analysis. You have in-depth research on the impact of preoperative and intraoperative clinical indicators on postoperative AKI. Please analyze the correlation between changes (increased or decreased) in the following clinical indicators during the surgery and the risk of postoperative AKI, and explain their biological mechanisms. If the role of any clinical indicator is unclear, please mark it as 'uncertain.' The following is the list of clinical indicators:

**Chatgpt 4.5：**Elevated creatinine levels are indicative of impaired kidney function. The kidneys are responsible for filtering creatinine from the bloodstream, so when they are not functioning properly, creatinine levels rise. This elevated creatinine reflects a reduced ability of the kidneys to clear waste, which can increase the risk of acute kidney injury (AKI). In the context of surgery, elevated creatinine levels suggest that the kidneys are under stress or experiencing dysfunction, raising concerns for postoperative AKI.

**Chatgpt 4o：** Similar to the previous response, increased creatinine levels serve as a marker for renal dysfunction. When kidney function is compromised, creatinine, a byproduct of muscle metabolism, accumulates in the blood. This increase is often an early warning sign of potential AKI, which can be exacerbated by factors such as blood loss, reduced blood pressure, or medications used during surgery that affect renal function. Therefore, elevated creatinine levels during or after surgery can point to the onset of AKI.

**Google Gemini 2.5：**Elevated creatinine is directly associated with a reduction in the glomerular filtration rate (GFR), which is the measure of how well the kidneys are filtering blood. When the GFR drops, it indicates that the kidneys are not efficiently removing waste products from the blood, leading to the accumulation of substances like creatinine. The increase in creatinine levels is a key diagnostic and prognostic tool in AKI, as it reflects kidney dysfunction, and tracking this marker can help in diagnosing and staging AKI in postoperative settings.

**DeepSeek-R1:70B：**Creatinine is considered a direct and reliable marker of kidney function. In the event of kidney injury or dysfunction, such as that seen in AKI, the kidneys are unable to filter creatinine effectively, resulting in higher serum creatinine levels. Elevated creatinine levels are commonly used to assess kidney function and are strongly correlated with an increased risk of AKI. Monitoring creatinine levels during and after surgery is essential for identifying patients at risk for kidney injury.

**Gemma 3 27B：** Elevated creatinine levels indicate a deterioration of kidney function. This worsening renal function can be a result of various factors such as dehydration, decreased blood flow to the kidneys, or surgical complications. When creatinine levels rise, it suggests that the kidneys are not filtering waste as efficiently, and this is a strong indicator of AKI. A significant increase in creatinine during surgery is an important warning signal that AKI may develop in the postoperative period.

**Qwen 3 30B：** Creatinine is a well-established marker of kidney function, and an increase in its levels during surgery suggests that the kidneys are experiencing dysfunction. This could be due to a variety of factors, including blood loss, medication effects, or compromised blood flow. An elevation in creatinine is a direct indicator of worsening renal function, which is a critical sign of AKI. Monitoring creatinine levels during surgery and the postoperative period helps identify patients at risk and guides early intervention strategies to prevent further kidney damage.

**(2) To eliminate hallucinations in AI, ask the LLMs again about the results:**

Please assess whether the clinical indicator names and their functional descriptions are reasonable. If they are, please respond 'Yes.' If not, please respond 'No' and provide the correct biological function of the clinical indicator, particularly its role in the occurrence of postoperative AKI. The specific input is as follows: Creatinine (serum)+ initial result.：

**Chatgpt 4.5：**Elevated creatinine levels are one of the most commonly used biomarkers to assess kidney function. The kidneys filter out creatinine from the blood and excrete it in the urine. When kidney function is compromised, as is the case in postoperative AKI, the kidneys' ability to clear creatinine decreases, leading to an accumulation in the blood. This elevated serum creatinine is a critical indicator for diagnosing AKI, particularly in postoperative patients who may experience kidney stress due to factors like hypotension, blood loss, or nephrotoxic medications. Therefore, monitoring creatinine levels post-surgery is essential for identifying renal dysfunction early, enabling timely intervention.

**Chatgpt 4o：**Serum creatinine is indeed a crucial marker for assessing kidney function. When kidney function declines, creatinine, a waste product of muscle metabolism, accumulates in the blood because the kidneys' filtration capacity is impaired. In the context of postoperative AKI, creatinine levels rise as a result of decreased renal clearance, which could be due to a variety of surgical-related factors like blood flow disruption, anesthesia effects, or use of medications that impact kidney function. Elevated creatinine levels are a primary indicator of kidney injury and are frequently used to monitor patients’ renal status after surgery, guiding management decisions to prevent further kidney damage.

**Google Gemini 2.5：** Creatinine is produced continuously in the body as a byproduct of muscle metabolism and is usually filtered out by the kidneys. Under normal circumstances, the kidneys maintain a relatively stable level of creatinine in the blood by excreting it in urine. However, when kidney function is compromised, as seen in postoperative AKI, the kidneys’ ability to filter out creatinine decreases, causing it to accumulate in the bloodstream. This rise in serum creatinine is directly correlated with the severity of kidney dysfunction and serves as an essential diagnostic tool for identifying AKI. Its increase can signal the progression of AKI and can help physicians assess the severity of kidney injury following surgery.

**DeepSeek-R1:70B：**The serum creatinine level is one of the most reliable biomarkers for assessing kidney function. It is widely used in clinical practice to diagnose and monitor kidney injury, including AKI in postoperative settings. When renal function is impaired, creatinine is not cleared as efficiently, and its concentration in the bloodstream increases. In the case of postoperative AKI, factors such as hypotension, ischemia, or nephrotoxic medications can contribute to kidney injury and lead to a rise in creatinine. Therefore, serum creatinine is a valid and essential marker for identifying AKI in postoperative patients, helping healthcare professionals track changes in renal function and intervene appropriately.

**Gemma 3 27B：**Creatinine is a waste product that is normally filtered by the kidneys. When kidney function is normal, it is excreted through the urine. However, in postoperative AKI, kidney filtration is impaired due to factors like decreased blood flow or direct injury to the kidneys during surgery. This results in higher serum creatinine levels. Elevated creatinine levels indicate a reduced ability of the kidneys to filter waste, making it a significant indicator for AKI. Monitoring creatinine post-surgery is critical, as it helps identify renal impairment and is used to diagnose and stage the severity of AKI. In postoperative patients, timely detection of elevated creatinine levels allows for early intervention to manage and mitigate AKI risk.

**Qwen 3 30B：**Yes. Serum creatinine is a key indicator of kidney function. Elevated levels reflect impaired renal clearance, as creatinine is a waste product filtered by the kidneys. In acute kidney injury (AKI), reduced glomerular filtration rate (GFR) leads to decreased creatinine excretion, causing its accumulation. This makes serum creatinine a reliable biomarker for detecting and monitoring AKI, though it may lag behind early kidney damage due to its dependence on muscle mass and other factors.

1. **Finally, the following conclusion was drawn after merging through the latest ChatGPT 5 model:**

While serum creatinine is a crucial marker in diagnosing and monitoring AKI, it is essential to recognize its limitations in the early stages of kidney injury, particularly when the rise in creatinine may not be immediate. This delay is due to the fact that creatinine levels depend on variables such as muscle mass and hydration status, which can obscure its ability to detect subtle or early renal dysfunction.

However, elevated serum creatinine remains one of the most widely used biomarkers for assessing kidney function and remains integral in both the clinical diagnosis and prognosis of AKI, particularly in postoperative settings where renal stress is common. Its role in identifying kidney impairment and guiding clinical decision-making cannot be understated, but clinicians should also be aware of other diagnostic tools and biomarkers that may offer earlier detection of kidney injury.

In conclusion, while serum creatinine is a reliable and widely accepted indicator for detecting AKI, its limitations in detecting early-stage kidney dysfunction highlight the importance of incorporating additional diagnostic methods and continuous monitoring in high-risk patients. This approach will allow for more timely interventions and improved outcomes in patients with postoperative AKI.
